# Supplementary material for: Are carbon emissions trading and green financial instruments synergistic? -Comprehensive quantitative research based on content analysis
Source: PLoS One. 2024 Mar 7;19(3):e0298601. doi: 10.1371/journal.pone.0298601 (PMC10919601; doi:10.1371/journal.pone.0298601)
Supplement: S1 Data — (ZIP) [file pone.0298601.s001.zip › Data/地级市Z_F工作报告.zip!/╔┬╬≈/╧╠╤⌠╩╨/└┤╫╘╣1⁄2╓┌║┼í╛┬φ┐╦╩2╛▌═°í┐_2014.docx]

各位代表：

　　　　现在，我代表市人民政府向大会作工作报告，请予审议，并请市政协委员和其他列席人员提出意见。

　　　　一、2013年工作回顾

　　　　过去一年，在省委、省政府和市委的坚强领导下，我们深入贯彻党的十八大精神，牢牢把握“稳中求进、好中求快”的总基调，以共建大西安为统揽，解放思想，真抓实干，全市经济继续保持在稳定上升通道，圆满完成市七届人大一次会议确定的目标任务，实现了新一届政府的良好开局。主要做了八个方面工作：

　　　　（一）加强研判，综合施策，经济保持平稳较快增长。面对经济下行压力加大的复杂形势，坚持每季度提前一个月进行分析预判，及时解决苗头性问题，牢牢掌握发展主动权。加强经济运行调节，强化财政调动作用，落实工业发展专项资金1亿元；发挥金融杠杆撬动作用，各类金融机构新增贷款116亿元；注重科技研发推动作用，科技研发投入8.3亿元、技术合同交易额23.2亿元。坚持对标管理，认真学习省内外先进经验，以目标责任考核推进落实。全市生产总值完成1860.4亿元、增长13.1%，高出全省2个百分点；财政总收入、地方财政收入分别完成217.9亿元、79亿元，增长3.7%、22.3%；城乡居民收入分别达到28488元、8538元，增长10.6%、14.4%。

　　　　（二）壮大产业，优化结构，转变发展方式迈出新步伐。致力于打基础、利长远，加快产业结构调整，提高经济运行质量和效益。积极应对冬春连旱，加快现代农业园区建设，促进农产品就地转化，食品工业增长27.6%。全年粮食产量189.4万吨，果、菜总产分别达到545.8万吨、387.3万吨，畜禽产品产量101.2万吨，农业生产总值稳居全省第一。扎实推进工业发展“1113”工程，装备制造、医药分别增长19.6%、11.6%，新材料、新能源产业占比提高到12.5%。规模工业增加值完成835亿元、增长18.7%。出台支持高新区发展意见，高新区二次创业步伐加快。加快新兴纺织工业园建设，企业搬迁和项目引进工作有序推进。列支3000万元支持非公有制经济发展，非公有制经济占比达到50.79%。实施振兴服务业三年行动计划，高科技历史文化产业园、苏宁云商西北电子商务运营中心等一批重大项目落户咸阳，乡村旅游发展活跃，文化产业增加值增长29%，社会消费品零售总额完成459.7亿元、增长15.4%。

　　　　（三）招商引资，项目带动，发展后劲持续增强。坚持战略招商，引进合同项目303个，合同引进资金2332亿元，到位资金708.1亿元。健全项目推进机制，150个市级重点项目完成投资765.3亿元，占年计划的126.3%。西咸新区50个项目完成投资159.9亿元，占年计划的121.1%。交通设施完成投资73.7亿元，西平铁路咸阳段全线建成，咸旬高速、西咸北环线加快推进，新建农村公路281公里。水利设施完成投资22.2亿元，东庄水库、羊毛湾水库供水工程顺利开工，亭口水库、红岩河水库、渭河综合治理工程进展良好，解决了56.6万人饮水不安全问题。全社会固定资产投资完成2054.5亿元、增长27.1%。

　　　　（四）全域谋划，统筹推进，城乡一体化步伐加快。加快融入大西安，公交线路与西安地铁一号线及时对接，秦汉大道、正阳大道等13个道路对接工程全面开工。实施主城区改造提升“3+6”工程，140个年度城建项目进展顺利。打通文林西路、经电路等12条断头路，城西快速干道建成通车，路网结构得到优化。咸阳古渡、清渭楼南广场免费开放。北塬新城总体规划编制完成，市民文化中心等项目开工建设。启动全国文明城市创建工作，国家园林城市通过专家组考察评审，我市被确定为国家首批创建智慧城市试点市。深入实施县城三年大提升计划，彬县、淳化被评为国家卫生县城，省市重点示范镇完成投资24.4亿元，全市进城落户农民20.5万人。

　　　　（五）铁腕整治，多措并举，生态环境建设力度加大。扎实开展“治污降霾·保卫蓝天碧水”行动，总投资17.2亿元的10大类537个大气污染防治项目进展顺利，完成加油站油气回收治理工作，拆除市区燃煤锅炉147台，淘汰黄标车及老旧车辆5843辆，大气污染的突出问题得到有效遏制。渭河污染治理三年任务两年完成，彻底结束了污水直排渭河、泾河的历史。二氧化硫、氮氧化物、化学需氧量、氨氮排放量分别下降4.98%、7.04%、2.66%、6.24%，超额完成了计划任务。全年造林42.5万亩，绿化道路405.9公里，改造、新增城市绿化面积13.3万平方米。发展节水灌溉18万亩，治理水土流失506.3平方公里。扎实开展农村环境连片整治工作，建成省市级生态示范村45个。

　　　　（六）以人为本，民生优先，群众生活进一步改善。坚决落实“两个80%”的硬指标，70类655个民生项目完成投资113.6亿元，占年度计划的133.2%。新增城镇就业6.3万人，城镇登记失业率控制在3.1%。学前教育三年行动计划全面完成，累计新建扩建公办幼儿园196所。“双高双普”工作全面启动，义务教育营养改善计划和“蛋奶工程”覆盖面达到82.9%。公立医院改革深入推进，群众住院“先看病、后付费”模式在基层医疗卫生机构全面推广。出生人口素质不断提升，人口自然增长率控制在4.72‰。启动实施城镇职工、居民大病二次报销，城镇居民医疗保险、新农合补助标准均提高到300元，城乡居民基础养老金标准提高到85元。新增养老床位4725张，为城乡低保户发放低保金6.4亿元。建设广播电视户户通6.3万套，成功举办中国文化遗产日主场城市活动。新开工保障性住房29304套，完成投资64.5亿元，占年度计划的119%。争取5亿元国债资金，全面启动旱腰带区域特色城镇化移民搬迁工作，12.33万人稳定脱贫。

　　　　（七）健全机制，综合治理，社会大局和谐稳定。扎实推进平安建设十大工程，加强对重点人群和特殊人群的帮教管理，严厉打击各类犯罪，社会治安满意度和平安建设知晓率不断提升。持续开展“六五”普法，依法治市取得新进展。建立群众矛盾诉调对接机制，集中开展信访积案化解活动，一批信访问题得到有效解决。食品药品监管机构改革稳步推进，监管能力进一步提升。从严排查整治事故隐患，安全生产形势平稳，被评为全省安全生产先进市。

　　　　（八）转变作风，优化服务，政府效能建设得到加强。认真执行市人大及其常委会决定，及时听取人大代表、政协委员的意见和建议，共办理市人大代表建议209件、市政协委员提案625件。以建设“五型”政府为目标，严格执行中央“八项规定”，精简会议文件，规范节庆论坛，清理办公用房，整顿公务用车，“三公”经费同比下降9.6%。下放精简行政审批380项，投资发展环境“三项整治”活动扎实开展。完善应急管理机制，突发事件处置能力得到加强。扎实开展“访百企、进千村、入万户”群众路线调研活动，为群众解决问题4162件。

　　　　双拥、老龄、慈善、残疾人、红十字等事业全面发展，外事、侨务、对台、审计、统计、民族、宗教、工商、人防、气象、地震、地方志等工作有了新进步。

　　　　各位代表，一年来的工作实践，使我们深刻感受到，咸阳的每一步发展，政府的每一项工作，离不开省委、省政府和市委的坚强领导，离不开市人大、市政协的监督支持，更离不开全市人民的艰苦奋斗，这是我们实现“咸阳梦”的根本保障和动力源泉。在此，我代表市人民政府向全市人民，向驻咸部队、武警消防官兵和政法干警致以崇高的敬意！向各位人大代表、政协委员和所有关心咸阳发展的各界人士表示衷心的感谢！

　　　　在肯定成绩的同时，我们也清醒地认识到发展中存在的问题和不足，主要是：经济总量偏小，欠发达的市情还没有得到根本改变。经济结构不合理，战略性新兴产业占比小，服务业发展相对滞后，科技贡献率低，转变发展方式刻不容缓。重大产业项目尤其是带动力强的工业大项目比较少，投资后劲不足。资源环境约束日益趋紧，治理大气污染任务艰巨。城乡居民收入渠道不宽，增长相对较慢。安全生产工作基础不够牢固，影响社会稳定因素比较多。干部作风需要进一步转变，勤政廉政建设和反腐败斗争仍然是长期任务。对这些困难和问题，我们不回避、不懈怠，将采取措施认真加以解决。

　　　　二、2014年工作目标

　　　　2014年是深入贯彻落实党的十八届三中全会精神、全面深化改革的首战之年，也是实施“十二五”规划的攻坚之年。总体判断今年形势，机遇大于挑战、动力强于压力。从宏观看，我国经济虽然正处在增长速度换挡期、结构调整阵痛期、前期刺激政策消化期，不稳定不确定不可持续的因素较多，但长期向好的基本面没有改变。从我市看，咸阳已进入“四化”同步发展的新阶段，一些重大规划和重点项目正在加快实施，新的经济增长点加速形成，经济持续健康发展的基础牢固。随着中央深化改革、推进新型城镇化、建设丝绸之路经济带等重大政策的密集出台，大西安建设提速、西咸新区成为国家级新区、我市入列全国资源成长型城市，咸阳的发展机遇前所未有，完全有基础、有条件把经济发展的良好势头保持下去。

　　　　综合以上分析，咸阳今年及今后一个时期的发展，不是防止“失速”的问题，而是抢抓机遇、发挥优势，全力打造经济升级版，实现更好更快更高质量发展的问题。为此，确定今年经济社会发展的主要预期目标是：生产总值增长13%左右，全社会固定资产投资增长25%，规模以上工业总产值、增加值均增长16%，社会消费品零售总额增长15%，地方公共财政预算收入增长14%，城镇居民人均可支配收入增长11%，农村居民人均纯收入增长12%，单位GDP能耗下降3.3%，二氧化硫、氮氧化物、化学需氧量、氨氮排放量分别削减0.5%、6%、1%和1%。

　　　　围绕上述目标，我们将全面贯彻落实党的十八大和十八届三中全会、中央经济工作会议、省委十二届四次全会和市委六届八次全会精神，坚持“稳中求进、改革创新”的工作基调，坚定信心，奋力攻坚，在“稳增长、调结构、抓改革、促开放、优环境、惠民生”上下功夫、求突破，着力提升发展质量效益，着力保持快速增长态势，着力增强发展活力后劲，着力提高群众幸福指数。我们坚信，只要全市上下矢志不移抓转型、凝心聚力促发展，就一定能够开创共建大西安、实现咸阳梦的新局面！

　　　　三、2014年工作重点

　　　　（一）大力发展现代农业，促进农民稳定增收。认真贯彻中央一号文件精神，以促进农民增收为核心，创新农业生产经营体系，加快推进传统农业向优质高效、生态观光农业转变，努力实现农业强、农民富、农村美。

　　　　坚持抓基地、增效益。实施粮食高产创建、果业提质增效、畜牧规模扩张、蔬菜设施栽培等十大工程，加快500万头PIC生猪基地和粮区30万头高产奶牛、城郊千万只肉鸡等项目建设，新建省市级现代农业园区30个，发展优质出口苹果25万亩、设施蔬菜3万亩。加强耕地保护，改造中低产田，确保粮食播种面积590万亩以上，总产保持在180万吨。

　　　　坚持抓销售、创品牌。完善农产品销售网络，实施批发市场信息化提升工程，壮大农民经纪人队伍，推进农超对接，拓展网上交易。制定农业品牌培育计划，提升咸阳苹果、蔬菜、手织布影响力。

　　　　坚持抓龙头、促增收。健全农业社会化服务体系，促进农民专业合作经营组织规范发展，建成市级百佳示范社30家。培育引进龙头企业，支持双汇、温氏、雨润等企业发展，带动家庭农场、专业大户等发展壮大。深化咸·杨农业产业一体化合作，加大科技推广力度，提高农业综合效益。推广袁家村、马嵬驿发展模式，带动乡村旅游发展，打造大西安休闲避暑的后花园。

　　　　坚持抓管理、保质量。注重源头管理，将农产品质量安全纳入县镇政府绩效考核范围，加强农产品质量安全监管检测机构建设，创建省市级农业标准化示范区15个，建立“从田间到餐桌”的食品安全保障体系，确保广大人民群众吃得好、吃得安全。

　　　　坚持抓基础、强保障。加快农业机械化示范区建设，提升农业机械化水平。创新土地经营权流转管理服务方式，发展适度规模经营。实施羊毛湾水库供水、亭口水库、红岩河水库、张家山泉群开发等工程，做好东庄水库、引汉济渭保障服务工作，发展节水灌溉16万亩，治理水土流失450平方公里，提高农业抗御自然灾害的能力。

　　　　（二）强化工业主导，加快转型升级步伐。坚持把工业作为稳增长、调结构的主战场，着力在优化布局、壮大支柱、提升效益上下功夫，不断增强工业经济竞争力。

　　　　以稳产促销为重点确保工业持续增长。强化运行调节和要素保障，足额落实工业发展基金，继续开展“送政策、送服务、送支持、送信心”活动，认真解决煤炭、水泥等行业产品销售问题，以销促产、以销稳产。

　　　　以“1113”工程为抓手提升整体实力。列支3000万元贷款贴息支持100个扩能技改项目，加大对100户成长型企业扶持力度，推动100个重点工业项目早日建成投产，确保30户大企业大集团实现工业总产值1000亿元。

　　　　以园区建设为载体推动聚集发展。严格落实支持高新区发展意见，促进高端产业和创新要素向高新区聚集，打造支撑区域发展增长极。制定信息产业规划，支持中电彩虹集团加快发展，围绕三星闪存芯片项目发展配套产业，振兴电子信息产业。加快新兴纺织工业园建设，推进纺织企业退城入园，发展下游配套产业，促进纺织产业转型升级。规划建设清洁能源基地项目，带动石油炼化、煤化工产业聚集发展。加快医药产业园建设，促进医药产业集群化发展。规划建设汽车产业园，积极对接上汽集团、华晨集团，培育发展汽车整车生产及配套产业，壮大装备制造业。加快新能源产业园建设，着力培育新的增长点。

　　　　以民营经济为支撑壮大市场主体。实施重点民营企业培育工程，制定民营企业进入特许经营领域具体办法，鼓励民间资本进入交通、能源、金融、市政、医疗卫生、公共服务等行业，支持民营企业通过参股、购买、租赁等形式参与国企改制和经营。列支3000万元扶持中小企业发展，设立1000万元中小企业信贷风险补偿金，鼓励组建融资互助合作组织，推动企业在多层次资本市场直接融资，支持民营企业做大做强。

　　　　以科技创新为龙头加快“两化”融合。围绕创建国家创新型试点城市，实施“12351”工程，加快陕西科技资源中心咸阳分中心建设，促进院企、校企合作，深化科技与金融结合试点，不断提升科技支撑引领作用。深化工业化、信息化“两化”融合，培育典型示范企业50户。实施“质量强市”战略，支持企业争创名牌。

　　　　（三）壮大现代服务业，释放内需拉动作用。全力推进振兴服务业三年行动计划，促进服务业发展提速、比重提高、水平提升。

　　　　大力发展生产性服务业。依托区位优势，规划建设电子商务产业园，积极招引大型物联网企业、知名电子商务龙头企业，加快天士力（陕西）医药物流中心、空港国际物联城等项目建设。实施宽带咸阳战略，加快4G网络建设，推进宽带下乡、光网入户，扩大信息消费。培育壮大科技咨询、商务会展、金融服务等产业，提升生产性服务业专业化水平。

　　　　着力打造文化、旅游、养生三大特色品牌。培育文化市场主体，加快文化产业孵化基地建设，推进文化与旅游、金融、科技融合发展。全力抓好高科技历史文化产业园、乾陵唐文化大景区等项目建设，支持红色旅游、生态旅游加快发展，打造一批乡村旅游名镇名村。培育壮大养生保健、休闲养老等健康产业，提升中国第一帝都、中华养生名城的美誉度和吸引力。

　　　　不断扩大城乡消费。以建设大西安商贸副中心为目标，加快红星美凯龙咸阳店、五洲国际·西北商贸博览城等项目进度，推进中央商务区、商业功能区和特色商业街区建设，着力发展一批商贸示范县和商贸重镇，打造便民消费圈，活跃城乡消费。

　　　　（四）强化投资拉动，着力增强发展后劲。继续坚持战略招商、项目带动，以投资结构优化提升经济增长效益。

　　　　狠抓重点项目建设。今年150个重点项目总投资4523.5亿元，年度投资689亿元。严格落实“五个一”工作机制，建立健全准入退出、效益评估以及定期协调会议制度，做好正泰智能电气西北产业园等项目前期工作，加快180万吨煤制甲醇、现代农机装备制造产业园等项目建设。

　　　　提升招商引资效益。建立重大产业项目前期策划及前置评估制度，充实重大产业项目库。继续实行“一套班子外出抓招商、一套班子在家抓落实”机制，紧盯世界500强、央企、民营100强，有效开展点对点招商、产业链招商、委托中介招商。坚持招商委员会会议制度，强化服务保障，确保招商引资到位资金突破750亿元。

　　　　优化投资发展环境。推进集约节约用地，设定土地投资强度，清理低效闲置土地，深化城乡建设用地增减挂钩试点，提高土地利用效益。继续开展优化环境“三项整治”活动，依法严肃查处干扰项目建设和企业生产的不法行为，让企业和投资者安心、放心、专心发展。

　　　　（五）坚持区域联动，打造开放开发新高地。抓住国家扩大内陆地区对外开放的机遇，发挥承东启西的区位优势，以主动融入大西安为突破口，进一步提升经济外向度。

　　　　全力共建大西安。加强与西安在规划、基础设施等方面的对接，加快西咸北环线工程建设，力争地铁一号线二期工程早日开工。深化两市在产业发展、环境治理等方面的合作，着力建设大西安制造业配套基地、能源建材基地、科技成果转化基地、生产性服务业基地和农副产品供应基地。全力服务西咸新区建设，做好50个重点项目征地拆迁工作，支持沣西新城信息产业园、泾河新城中国锂产业园、空港新城综合保税区以及秦汉新城有色光电项目建设，实现一体共赢发展。

　　　　积极参与丝绸之路经济带建设。加强与丝绸之路沿线城市在能源转化、食品、纺织、旅游等领域的合作，支持企业拓展中亚市场，打造东部产品中转西进和西部地区、中亚国家资源深度转化的黄金通道。做好丝绸之路申遗工作，加快建设秦汉文化交流展示基地。建设泾阳茯茶古镇，打造西部茯茶加工贸易中心。

　　　　大力发展县域经济。优化县域产业布局，提升县域工业集中区建设水平，着力打造咸兴武高新技术和装备制造、彬长旬能化、乾礼泾现代建材、兴礼泾三食品等工业板块，支持秦都、渭城壮大城市经济，鼓励永寿、淳化建设绿色食品生产基地。深化争先进位活动，激发县域经济发展活力。

　　　　（六）加快城镇化进程，促进城乡一体发展。坚持“以人为本、四化同步、科学布局、绿色发展、文化传承”原则，积极稳妥推进新型城镇化。

　　　　优化城乡规划布局。按照“一核三带多点”空间布局，加快编制中心城市总体规划，完善城镇群、县域及村镇规划，引导城镇合理分工、功能互补，促进产城融合发展。加快北塬新城核心板块开发建设，推进中心城市与高新区、纺织工业园基础设施对接，优化城市生产、生活、生态空间结构。

　　　　提升城镇发展质量。继续推进中心城市“3+6”工程，精心谋划建设一批城建项目，加快主要出入口改造、人民广场、高铁咸阳站广场建设，完善供热、供气、供水和雨污水管网。抓好文兴路、秦皇路北段改造提升等工程，编制地铁发展规划，构建立体交通体系。加快智慧城市建设，推进城市智能化、精细化管理。开展美丽县城和美丽城镇建设，以16个省市重点示范镇、旅游文化名镇为重点，完善公共服务设施，实施一批国省干线过境段升等改建工程，提升城镇承载力和带动力。深入推进文明社区、文明村镇等创建活动，扎实开展全国文明城市创建工作。深化“多城同创”，多栽树、多蓄水、多种花，加强文化遗存保护、传承和利用，让城镇延续历史、融入自然，让市民舒心居住、健康生活。

　　　　推进城乡一体化发展。编制完成《城乡发展一体化战略规划》，探索建立以工促农、以城带乡、工农互惠、城乡一体发展机制。全面放开县城、建制镇落户限制，认真解决农民工子女上学、社会保障问题，让农民工进得来、留得住。支持农村居民就地就近市民化，年内进城落户农民15万人。加快新型农村社区建设，让农村成为幸福家园、美丽乡村。

　　　　（七）保护生态环境，加快建设美丽咸阳。把环境保护工作作为科学发展的“生命线”，一手抓治理，一手抓再造，不断改善生态环境。

　　　　全力打好治污降霾攻坚战。扎实开展“治污降霾·保卫蓝天碧水”行动，实施总投资17.8亿元、14类511个治污降霾重点项目，抓好燃煤锅炉、火电、水泥等行业治污设施提标改造。加强渭河、泾河流域水污染综合治理，推进10个新建污水处理厂项目按期达标运行，确保渭河水质三年变清目标如期实现。

　　　　全面加强生态建设。以关中大地园林化工程为抓手，加快建设渭河生态景观轴和五陵塬风光带，造林25万亩，发展杂果经济林10万亩。以“清洁水源、清洁家园、清洁田园、清洁能源”为目标，扎实开展综合整治，全面改善农村环境。

　　　　健全生态文明机制。启动主体功能区规划编制工作，优化国土空间开发格局。加强监管监测能力建设，实施水、气环境质量补偿考核机制，调动市场力量促进资源节约和生态保护。积极倡导低碳出行、绿色消费，让保护环境成为每个人的自觉行动。

　　　　（八）加强财税金融工作，提升发展支持保障能力。

　　　　强化财政保障作用。抓好收入征管，健全税源信息管理、税收征管和财税联合稽查机制，加强非税收入管理，坚决杜绝跑冒滴漏、收过头税，确保财政收入稳定增长。紧盯中省政策，强化项目申报，争取更多中省资金，持续扩大总财力。深化市、县两级部门预算和“三公”经费公开改革，从严控制一般性预算支出。整合专项资金，最大限度地带动金融资本、社会资金，优化财政支出结构，提高财政资金使用效益。建立政府举债融资和风险预警机制，将债务收支纳入预算管理，规避财政风险。

　　　　提升金融服务水平。加快完善金融市场体系，支持金融创新，实施“五个一”工程，强化金融服务实体经济能力。优化金融机构网点布局，发展新型金融组织。健全金融机构支持经济发展奖励机制，推进银、政、企合作。加强融资担保体系建设，强化城投公司、担保公司融资功能。积极争取世行贷款项目。支持企业开展直接融资，建立企业上市培育机制。优化金融生态环境，健全社会信用体系，打造诚信咸阳。

　　　　（九）实施民生工程，不断提高群众幸福感。按照“保基本、兜底线、促公平、可持续”的原则，严格落实改善民生“两个80%”制度，真正把财政资金用到群众最需要、最关注的地方。

　　　　实施就业优先战略。落实扶持创业优惠政策，加大小额担保贷款支持力度，加强职业技能培训，扎实做好高校毕业生、城镇失业人员、农村转移劳动力等重点群体就业工作，力争全年城镇新增就业5万人，城镇登记失业率控制在4.2%以内。

　　　　健全社会保障体系。按照“应保尽保、应发尽发”的要求，扩大社会保障覆盖面，不断提高经办服务水平。将城镇居民医保和农民新农合政府补助标准提高到350元，开展城乡重特大疾病医疗救助，完善城乡低保与临时困难救助、医疗救助相统一的社会救助体系。继续抓好安居工程，确保保障房公开公平公正分配。

　　　　大力发展社会事业。坚持教育优先战略，深化教育领域综合改革，抓好“双高双普”工作，大力发展职业教育，积极推进素质教育，促进教育均衡协调发展。坚持规划优先、资源共享，推动学校、企事业单位的文化、教育、体育等设施向群众开放。加快市民文化中心等基础设施建设。全力做好省第十六届运动会筹备工作。深化县级公立医院综合改革和县镇医疗服务一体化，提升医疗卫生服务水平。扎实推进母亲健康、免费孕前健康检查、计生家庭创业三大工程，人口自然增长率控制在5.6‰以内。大力发展养老服务，支持社会力量兴办养老服务机构。抓好双拥共建工作，争创“全国双拥模范城市”七连冠。

　　　　扎实推进扶贫开发。以旱腰带、六盘山片区为重点，坚持整体规划和精准到户相结合，跨镇村规划、集中连片开发，完成旱腰带区域特色城镇化移民搬迁工程任务，稳定脱贫10万人以上。

　　　　不断创新社会治理。坚持系统治理、依法治理、综合治理、源头治理，制定出台社会治理五年规划。深入推进平安咸阳建设，依法打击各类违法犯罪活动。推广“枫桥经验”，创新群众工作方法。强化网格化管理，加快综合服务管理平台建设，加强流动人口和特殊人群的服务管理，对失业、失亲、失独等人群给予更多的关爱，最大限度地减少不和谐因素。深化党政领导包抓信访积案化解活动，健全矛盾纠纷排查调处机制，不断拓宽群众利益诉求表达渠道。加强防灾预警体系建设，提高地震、气象灾害防御能力。健全基层监管体系，保障人民群众饮食用药安全。强化安全生产属地管理、行业监管和企业主体责任，实施“131久安工程”，健全事故隐患定期排查治理机制，严格责任追究，坚决杜绝重特大安全事故发生。

　　　　各位代表，群众的幸福感就是我们的成就感。今年市政府在全力抓好以上工作的同时，围绕群众交通出行、优化生态环境、改善居住条件、提升社会保障和医疗服务水平、满足群众文化生活需求、促进教育协调发展、方便群众生活等八个方面，重点办好35件便民惠民实事。尽管今年财政压力很大，但在民生投入上，我们绝不含糊、绝不欠账，一定尽最大努力，把事关群众切身利益的事情办实办好，让老百姓的日子越过越舒心、越来越红火。

　　　　四、深化重点领域改革

　　　　认真贯彻落实中央全面深化改革的决定和省委的实施意见，稳步有序推进各项改革，释放改革红利，催生发展动力。

　　　　（一）推进行政体制改革。深化行政审批制度改革，做好省上下放行政审批事项的承接工作，建立并公开行政审批目录清单。对政府各部门行政审批事项和非行政审批许可事项，能取消的一律取消，能下放的一律下放，能简化的一律简化，能网上办理的一律网上办理，能在政务大厅办理的一律在政务大厅办理。基本完成市县政府机构改革工作，深化事业单位分类改革，研究探索政府购买社会服务实施办法。加快扩权强镇步伐，赋予重点镇部分县级经济社会管理权限。提升县市区政务服务中心功能，加强镇村两级便民服务中心建设。

　　　　（二）深化经济体制改革。深化国有企业改革，完善国有资产监督管理体制，促进优势企业资本结构多元化，推动困难企业转换机制，加快解决已改制企业遗留问题。全面推进工商登记制度改革，将注册资本改为认缴制，实行先照后证，建立规范有序、宽进严管的市场监管体系。

　　　　（三）抓好社会领域改革。建立财政转移支付同农业转移人口市民化挂钩机制，逐步把符合条件的农业转移人口转为城镇居民。健全农村“三留守”人员关爱服务体系。制定“单独二孩”生育政策配套措施。全面深化农村改革，有序推进农村土地承包经营权、农村宅基地和居民房屋产权确权颁证。研究制定失地农民保障措施，依法保护被征地拆迁群众的合法权益，确保失地农民生计有保障、收入稳定增加。

　　　　五、努力建设人民满意政府

　　　　围绕建设“学习型、法治型、服务型、务实型、廉洁型”政府，加快职能转变，不断提高政府的公信力、执行力。

　　　　（一）始终坚持解放思想，开拓创新。扎实开展全员大培训活动，着力提升公务人员服务发展、服务企业、服务群众的能力，不断增强抓改革、谋发展、保稳定的本领。深入推进解放思想，学在新处，谋在深处，干在实处，以创新的思维谋划工作，用发展的办法解决问题。

　　　　（二）始终坚持民主决策，依法行政。自觉接受市人大及其常委会的法律监督、市政协的民主监督和社会的舆论监督，认真办理人大代表建议意见和政协委员提案，主动听取民主党派、工商联、无党派人士的意见。健全科学民主决策机制，完善重大行政决策程序。扎实推进政务公开，建成市、县、乡三级协同办公、并联审批系统，积极推动网络问政，确保群众的知情权、参与权、监督权。

　　　　（三）始终坚持为民务实，勤政廉政。把群众诉求作为第一导向，深入开展党的群众路线教育实践活动，深化“四风”和“庸懒散浮”专项治理，健全联系群众长效机制。把破解难题作为第一责任，围绕发展大事、民生难事、稳定要事，集中主要精力，突破瓶颈制约，不断开创各项工作新局面。把推动落实作为第一要求，坚持对标管理，加强效能监察，以扎实的作风确保各项任务完成。把清廉从政作为第一准则，认真落实党风廉政建设责任制，强化审计监督、财政监督，坚决压缩“三公”经费，坚决整治文山会海，坚决反对奢侈浪费，做到为民、务实、清廉。

　　　　各位代表，同志们，改革大潮风正劲，咸阳发展正当时。让我们在市委的坚强领导下，务实担当，主动作为，不断把各项事业推向前进，为实现“四强三领先”西部强市“咸阳梦”而努力奋斗！
